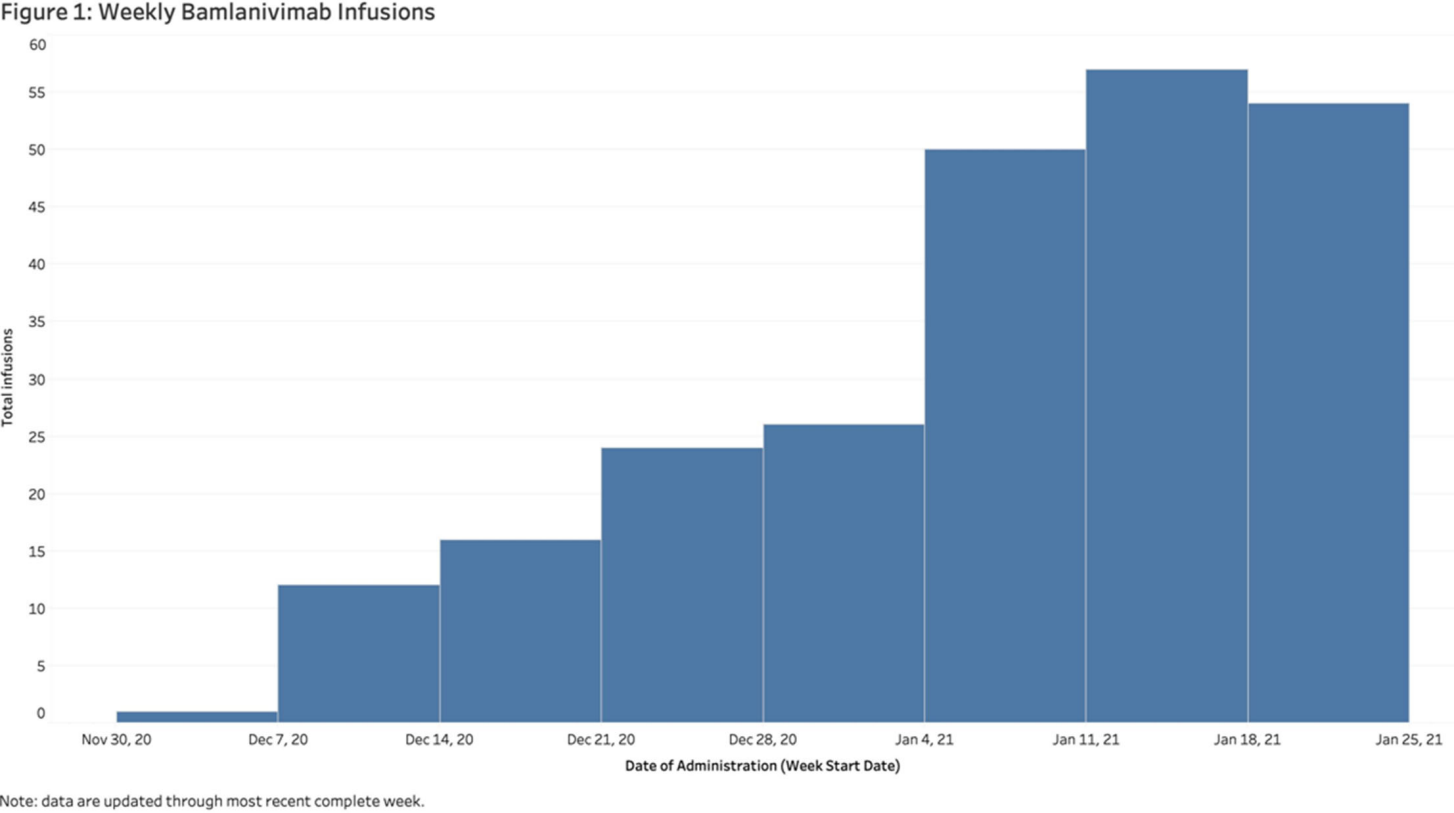# Antimicrobial Stewardship-Driven Monoclonal Antibody Treatment Program for COVID-19 Patients in the Bronx, New York

**DOI:** 10.1017/ash.2021.110

**Published:** 2021-07-29

**Authors:** Yi Guo, Victor Chen, Lauren Allen, Kelsie Cowman, Una Hopkins, Carol Sheridan, Edwin A Torres, Patricia Davis, Susan Sakalian, Frank Sosnowski, Priya Nori, Liise-anne Pirofski, Adam Haviland, James Rossi, Hongkai (Jack) Bao

## Abstract

**Background:** In November 2020, bamlanivimab received emergency use authorization (EUA) to treat patients with early, mild-to-moderate COVID-19 who are at high risk of progression. Montefiore Medical Center serves an economically underserved community of >1.4 million residents in the Bronx, New York. Montefiore’s antimicrobial stewardship team (AST) developed a multidisciplinary treatment pathway for patients meeting EUA criteria: (1) outpatients and hospital associates and (2) acute-care patients (EDs or inpatient). **Methods:** The Montefiore AST established a centralized process for screening high-risk COVID-19 patients 7 days a week. Referrals were sent by e-mail from occupational health, primary care practices, specialty practices, emergency departments, and urgent care centers. Patients were screened in real time and were treated in the ED or a newly established infusion center within 24 hours. After infusion, all patients received phone calls from nurses and had an infectious diseases televisit. Demographics, clinical symptoms, subsequent ED visit or hospital admission, and timing from infusion to ED or hospitalization were obtained from the electronic health record. **Results:** In total, 281 high-risk patients (median age, 62 years; 57% female) received bamlanivimab at the infusion center or in the acute-care setting between December 2, 2020, and January 27, 2021 (Table 1). The number of treated patients increased weekly (Figure 1). Also, 62% were Hispanic or black, and 96% met EUA criteria. Furthermore, 51 (18%) were referred from occupational health, 205 (73%) were referred from the community, and 25 (9%) were inpatients (https://www.fda.gov/media/143605/download). All patients were successfully infused without adverse reactions. In addition, 23 patients (8.2%) were hospitalized and 6 (2.1%) visited EDs within 30 days of treatment. The average number of days between symptom onset and infusion was 4.9. The median age of admitted versus nonadmitted patients was 68 years versus 61.5 years (*P* = .07). **Conclusions:** An AST-coordinated bamlanivimab treatment program successfully treated multiple high-risk COVID-19 patients and potentially reduced hospitalizations. However, the effort, personnel, and resources required are significant. Dedicated hospital investment is necessary for maximal success.

**Funding:** No

**Disclosures:** None

**Table 1.**
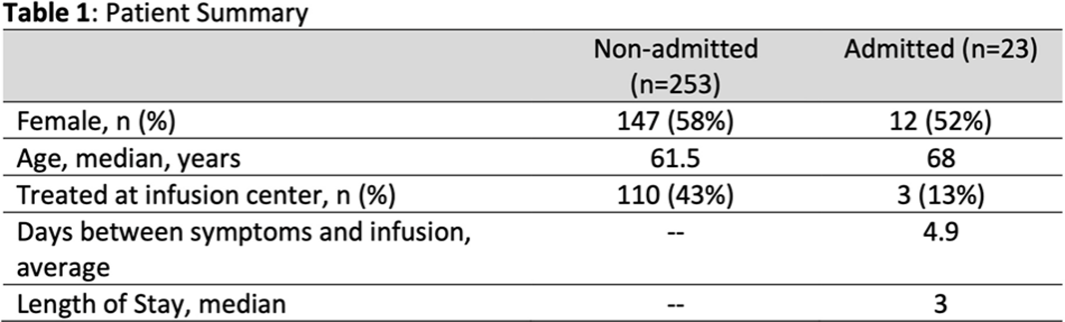

**Figure 1.**